# Co-parasitism of intestinal protozoa and *Schistosoma japonicum* in a rural community in the Philippines

**DOI:** 10.1186/s40249-018-0504-6

**Published:** 2018-12-10

**Authors:** Kosala Gayan Weerakoon, Catherine A. Gordon, Gail M. Williams, Pengfei Cai, Geoffrey N. Gobert, Remigio M. Olveda, Allen G. Ross, David U. Olveda, Donald P. McManus

**Affiliations:** 1 Molecular Parasitology Laboratory, Infectious Diseases Division, QIMR Berghofer Medical Research Institute, Brisbane, 4006 Australia; 2 School of Public Health, The University of Queensland, Brisbane, 4006 Australia; 3 Department of Parasitology, Faculty of Medicine and Allied Sciences, Rajarata University of Sri Lanka, Saliyapura, 50008 Sri Lanka; 4 School of Biological Sciences, Queen’s University Belfast, Belfast, BT9 7BL UK; 5 Research Institute for Tropical Medicine, Department of Health, Manila, 1781 Philippines; 6 Menzies Health Institute Queensland, Griffith University, Gold Coast, 4222 Australia

**Keywords:** Polyparasitism, Intestinal protozoa, Schistosomiasis japonica, The Philippines

## Abstract

**Background:**

Co-parasitism is a frequent occurrence in impoverished communities in the tropics resulting in a considerable disease burden. While there are extensive reports of intestinal helminthiases, including schistosomiasis japonica, the occurrence and extent of diseases caused by intestinal protozoa (IP) have yet to be investigated in depth in the Philippines. We present a detailed analysis of polyparasitism in a rural community of Northern Samar, focusing on co-infections of IP with *Schistosoma japonicum*.

**Methods:**

A descriptive cross sectional study was carried out in 2015 across 18 barangays (villages) endemic for *S. japonicum* in Northern Samar, the Philippines to assess the burden of human schistosomiasis and IP infections. Faecal samples collected from 412 participants from the 18 barangays were included in the final molecular analysis. A multiplex quantitative PCR assay was developed and used for the detection of *Blastocystis* spp., *Entamoeba histolytica*, *Cryptosporidium* spp. and *Giardia duodenalis* in stool samples. The findings were combined with previous results of droplet digital PCR diagnosis of individuals from the same 18 barangays infected with *S. japonicum* determined using the same stool samples for analysis.

**Results:**

Mean age of the study participants was 40.3 years (95% *CI:* 38.8–41.8) with 53% (*n =* 218) being males. Prevalence of *S. japonicum* (74.5%) and *Blastocystis* spp*.* (58.7%) was significantly higher compared to other infections, with *E. histolytica* having the lowest prevalence (12.1%). A majority of individuals were infected with more than one parasite with two infections being most common (*n =* 175, 42.5%). The prevalence of individuals with two parasites was significantly higher than all others with 27.9% (*n =* 115) subjects harbouring a single parasite species. Of individuals with two infections, *S. japonicum* and *Blastocystis* spp. were the most common combination (*n =* 110, 62.9%). Examining age within the population, 58.5% (*n =* 38) of school-aged children and 60.1% (*n =* 14) of women of child bearing age harboured at least two parasite species.

**Conclusions:**

The study revealed that polyparasitism with IP infections and schistosomiasis japonica is highly prevalent in individuals in Northern Samar which likely contributes to the significant public health and socio-economic burden suffered by this population. More generally, the findings are of relevance when considering implementation of integrated control strategies for intestinal parasites.

**Electronic supplementary material:**

The online version of this article (10.1186/s40249-018-0504-6) contains supplementary material, which is available to authorized users.

## Multilingual abstracts

Please see Additional file 1 for translations of the abstract into the five official working languages of the United Nations.

## Background

Concomitant intestinal parasitic worm infections, with water-borne intestinal protozoa (IP), occur frequently in impoverished communities and cause a serious public health burden with significant socio-economic impact [1, 2]. Many species of parasitic protozoa and intestinal helminths occur primarily in tropical zones due to the requisite shared environmental requirements of moist warm soil and water [1, 3, 4]. Aetiological factors that lead to infection with most intestinal parasites include poor socio-economic, sanitary and hygienic conditions and, with the schistosome blood flukes, the absolute requirement for suitable freshwater snail habitats and frequent human water contact [1, 3]. The high prevalence of these parasites and their considerable overlap in geographic distribution means that the potential for coinfection is high and likely to be more common than single infections in endemic areas [4–7]. Polyparasitism is thus widespread in endemic regions in the tropics, and the health impacts of co-infection can be more severe than when an individual is infected with a single parasite species [1, 5, 8].

It is estimated that schistosomiasis results in more than 40 000 deaths every year and some 700 million are at risk of infection in endemic areas [9, 10], whereas other intestinal helminths, for example the soil transmitted helminths (STH) (including *Trichuris trichiura, Ascaris lumbricoides* and the hookworms *Ancylostoma duodenale* and *Necator americanus*), infect more than one billion people worldwide [11]. Waterborne IPs cause substantial global morbidity and mortality with the most common and important species including *Entamoeba histolytica*, *Giardia duodenalis*, *Cryptosporidium* spp. and *Blastocystis* spp. Amoebiasis, caused by *E. histolytica*, one of the most deadly of the protozoa, alone accounts for 100 000 annual deaths around the world. Giardiasis is globally distributed and has a prevalence of around 30% in the developing world with a significant disease burden in poverty-related communities [12–14].

Pathogenic intestinal parasitic infections lead to significant nutritional deficiencies with gastrointestinal morbidity and mortality, particularly among immune-compromised individuals, pregnant women and children in developing countries [12, 15, 16]. Amoebiasis leads to diarrhoea, amoebic dysentery, colitis and hepatic abscess [12, 17]. Generally, *G. duodenalis* infection is self-limiting but it can also result in chronic disease with persistent diarrhoea leading to malabsorption and weight loss [18, 19]. *Cryptosporidium* also results in a self-limiting, asymptomatic infection but can lead to severe diarrhoea, particularly in immunocompromised people and in children [20–22]. *Blastocystis spp*. is a common anaerobic IP of humans but its pathogenicity is still under debate [23, 24].

While polyparasitism is highly prevalent in many endemic areas, limited large scale studies have been conducted in terms of assessing its true burden and impact on health in these regions [7, 25]. This could possibly be due to the lack of screening procedures with good diagnostic performance that allows simultaneous examination of an individual for the presence of IPs and helminths. As such, most studies have focussed on the identification of single rather than multiple parasites in infected individuals [26]. Moreover, the interpretation of the clinical picture becomes complicated as presenting manifestations often tend to be quite generalised and similar between intestinal parasites [25–27]. Whereas immunodiagnostic methods can lack specificity and microscopy-based techniques lack sensitivity and are reliant on the capability of the microscopist, modern DNA-based molecular diagnostic tools, such as quantitative polymerase chain reaction (qPCR) and droplet digital PCR (ddPCR), can be applied for highly sensitive concurrent detection of these parasitic infections [5, 28, 29]. The multiplex qPCR approach, in particular, is helpful in simultaneous screening of multiple parasites in a single clinical specimen and has the additional advantages of minimizing reagent costs and processing time [5, 30, 31].

*S. japonicum,* is highly endemic in the Philippines [5, 6, 32–34] and there are also reports of variable prevalence of IPs in both urban and rural communities [35–38], but there have been no in depth investigations of the occurrence and extent of waterborne, poverty-related protozoan infections. Here we present a detailed analysis, of IP infections in a rural community of Northern Samar province, the Philippines, with a particular focus on co-infections with *S. japonicum*.

## Methods

### Sample collection, processing and storage

**Fig. 1:**
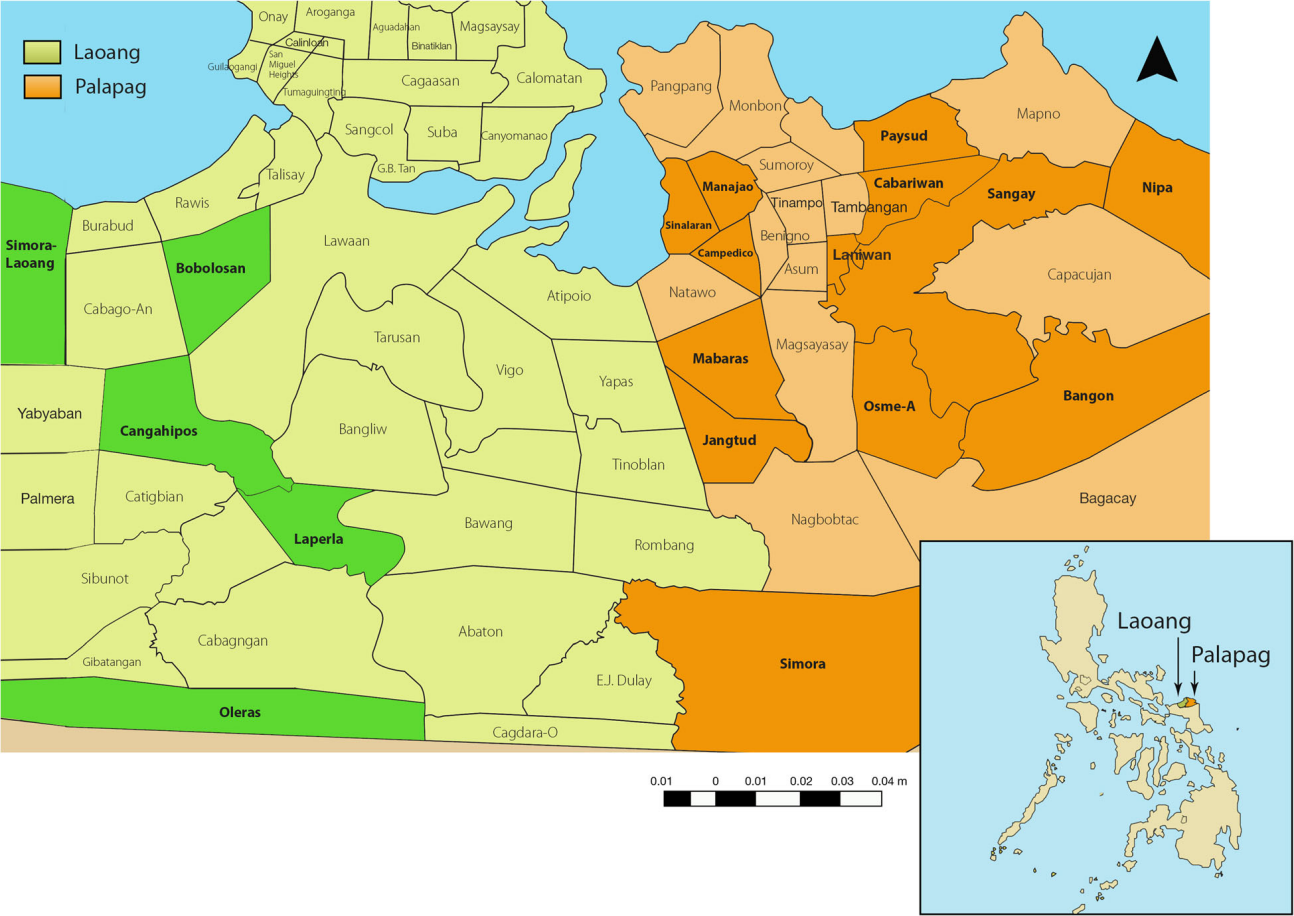
Map of the Philippines showing the Palapag and Laoang municipalities with the 18 study barangays. (Adapted from reference [40])

This descriptive cross-sectional study was carried out across eighteen barangays (villages) endemic for schistosomiasis japonica in Northern Samar (Palapag and Laoang municipalities), the Philippines (Fig. 1), to assess the burden of human schistosomiasis and IP infections. The study cohort has been described previously and the majority of the population in the area live below the poverty line and are faced with poor sanitation, lack of good hygiene practices, and limited water supply [32, 39]. The study area is known to be endemic for multiple parasitic diseases but the status of intestinal protozoan infections has not been systematically determined [5, 32, 39].

IP infection status (*Blastocystis* spp., *Entamoeba histolytica*, *Cryptosporidium* spp. and *Giardia duodenalis*) was assessed by a multiplex qPCR assay using DNA isolated from stool samples. Previous results of ddPCR diagnosis of *S. japonicum* infections using the same stool samples [40], were used for the analysis and comparison of infection status of *S. japonicum* with the IPs.

Stool samples were collected over a period of one week in August 2015. This study was conducted since 2012 as part of a large survey to evaluate the hepatic morbidity associated with schistosomiasis [32]. All participants who were followed up as a part of this hepatic morbidity investigation were considered in this study. Additionally, final inclusion into the current study was based on having a completed consent form and submitting a stool sample for analysis. A total of 452 participants from the 18 barangays were recruited for the study. Faecal samples were collected in a pre-labelled stool cup from each participant and age and gender were obtained at sampling. The Kato-Katz test was performed on faecal samples for the initial detection of *S. japonicum* infection and participants who were positive were treated with praziquantel. Around 10 g of faeces was fixed in 80% ethanol and stored at 4 °C for DNA extraction and PCR analysis carried out later at QIMR Berghofer Medical Research Institute (QIMRB) in Australia.

### DNA extraction

DNA isolation from faecal samples was performed using the Maxwell®16 Instrument (Promega Corporation; Wisconsin, USA) incorporating the Maxwell®16 LEV Plant DNA kit, as described previously [40]. Based on the adequacy of sample provided and successful DNA extraction, a final total of 412 samples were utilized in the subsequent molecular and data analysis.

### qPCR analysis

A multiplex qPCR was established employing primers and probes published earlier [30, 41] for *Blastocystis* spp., *E. histolytica, Cryptosporidium* spp. and *G. duodenalis* (Table 1). The total volume of 20 μl reaction mixture contained, 10 μl of GoTaq® Probe qPCR master mix (Promega Corporation, Madison, USA), optimised primer and probe concentrations (Table 1), and 2 μl of template DNA. Thermocycling conditions were 3 min at 95 °C with subsequent 40 cycles at 95 °C for 30 s, 55 °C for 30 s and 72 °C for 30 s in a Corbett RotorGene 6000 instrument (Qiagen, Hilden, Germany). No template DNA and positive controls were used in all assays. Positive controls included cultured plasmid DNA of *Blastocystis* spp., *E. histolytica, Cryptosporidium* spp., and *G. duodenalis,* which were kindly provided by the Clinical Tropical Medicine Group of QIMRB and the School of Veterinary Science, University of Queensland (Gatton Campus).

**Table 1:** Details of the multiplex qPCR primers and the probes used in the study

Parasite	Gene target	GenBank Accession #	Reference	Primer / probe	Sequence (5′ → 3′)	Product size (bp)	Final concentration (nmol/L)
*Blastocystis* spp.	SSU rRNA	AY244621	[41]	Forward primer	GGTCCGGTGAACACTTTGGATTT	119	350
				Reverse primer	CCTACGGAAACCTTGTTACGACTTCA		350
				Probe	FAM-TCGTGTAAATCTTACCATTTAGAGGA-MGBNFQ		120
*Entamoeba histolytica*	SSU rRNA	X75434.1	[30, 71]	Forward primer	AACAGTAATAGTTTCTTTGGTTAGTAAAA	135	200
				Reverse primer	CTTAGAATGTCATTTCTCAATTCAT		200
				Probe	ROX-ATTAGTACAAAATGGCCAATTCATTCA-IBRQ		80
*Giardia duodenalis*	SSU rRNA	M54878.1	[30]	Forward primer	GACGGCTCAGGACAACGGTT	63	200
				Reverse primer	TTGCCAGCGGTGTCCG		200
				Probe	CY5-CCCGCGGCGGTCCCTGCTAG-IBRQ		100
*Cryptosporidium* spp.	COWP	AF248743.1	[30]	Forward primer	CAAATTGATACCGTTTGTCCTTCTG	150	300
				Reverse primer	GGCATGTCGATTCTAATTCAGCT		300
				Probe	HEX-TGCCATACATTGTTGTCCTGACAAATTGAAT-IBFQ		75

*SSU rRNA* Small subunit ribosomal RNA, *COWP Cryptosporidium* oocyst wall protein

A serial 10-fold dilution of DNA extracted from each plasmid control was used to prepare a standard curve. The dilution series was used to optimize the multiplex qPCR assay and to set the cycle threshold (Ct) value cut-offs. Optimization was done as mono-assays of all four of the qPCRs (for *Blastocystis* spp., *E. histolytica, Cryptosporidium* spp., and *G. duodenalis*) and thereafter in the multiplex assay. The maximum Ct value considered to be positive was set at 35.

### Statistical analysis

Findings of the multiplex qPCR assay performed here were combined with our previous results of ddPCR diagnosis of *S. japonicum* infections using the same cohort stool samples [40]. Data analysis was done using Microsoft Excel 2010 (Microsoft; LA, USA), GraphPad Prism 7 (GraphPad Software, Inc.; California, USA) and R 3.4.0 (R foundation; Vienna, Austria) software. The Venn diagrams were designed using an online tool available at: http://bioinformatics.psb.ugent.be/webtools/Venn/, and then modified. Prevalence for all five parasites (*Blastocystis* spp., *E. histolytica, Cryptosporidium* spp., *G. duodenalis* and *S. japonicum*) was calculated from the total study sample. Infection intensities across age, gender and municipalities were analysed with Mann Whitney and Kruskal-Wallis tests for statistical significance. The chi-square (*χ*^2^) test was used to test the associations between prevalence of *S. japonicum* and the protozoa, and age, gender and municipalities. The odds ratio (*OR*) was used to assess the strength of association between multiple infections with a 95% confidence interval (95% *CI*). Statistical inferences were made with a significance level of 5% (*P =* 0.05).

## Results

### Description of study population

Of the total group (*n =* 412), 218 (53%) were male and the mean age of the entire sample was 40.3 years (95% *CI:* 38.8–41.8). The distribution of prevalence and intensity of the five parasitic infections in the total group, as well as across different municipalities, and age and gender categories are shown in Table 2.

**Table 2:** Prevalence and intensity of the intestinal protozoan and *S. japonicum* infections by gender, age, and municipality

Variables		Total		*Blastocystis* spp.					*Cryptosporidium* spp.					*Entamoeba histolytica*					*Giardia duodenalis*					*Schistosoma japonicum* ^a^				
		*n*	%	Positive	Prevalence		Ct score		Positive	Prevalence		Ct score		Positive	Prevalence		Ct score		Positive	Prevalence		Ct score		Positive	Prevalence		Intensity (CNI)	
					%	95% *CI*	Mean	95% *CI*		%	95% *CI*	Mean	95% *CI*		%	95% *CI*	Mean	95% *CI*		%	95% *CI*	Mean	95% *CI*		%	95% *CI*	Mean	95% *CI*
Total study sample		412	100.0	242	58.70	54.0–63.5	26.1	25.6–26.7	90	21.80	17.9–25.8	20.1	18.7–21.5	50	12.10	9.0–15.3	28.9	27.4–30.5	79	19.20	15.4–23.0	25.9	24.8–27.0	307	74.5	70.3–78.7	63.2	46.9–79.5
Gender	Male	218	52.9	121	55.5	48.9–62.1	27.1	26.3–27.8	42	19.3	14.0–24.5	19.4	17.5–21.4	29	13.3	8.8–17.8	28.4	26.0–30.7	46	21.1	15.7–26.5	26.4	24.9–27.8	163	74.8	69.0–80.5	91.7	62.2–121.2
	Female	194	47.1	121	62.4	55.6–69.2	25.2	24.4–25.9	48	24.7	18.7–30.8	20.7	18.7–22.7	21	10.8	6.5–15.2	29.7	28.0–31.5	33	17.0	11.7–22.3	25.3	23.5–27.0	144	74.2	68.1–80.4	30.9	23.9–37.9
Age	< 10 years	25	6.1	15	60.0	40.8–79.2	24.3	22.8–25.8	1	4.0	0.0–11.7	24.6	_	3	12.0	0–24.7	32.4	31.1–33.8	4	16.0	1.6–30.4	27.2	23.1–31.3	15	60.0	40.8–79.2	146.3	0–308.8
	10–18 years	40	9.7	24	60.0	44.8–75.2	27.6	25.6–29.5	12	30.0	15.8–44.2	21.0	16.4–25.6	4	10.0	0.7–19.3	31.9	31.1–32.7	3	7.5	0–15.7	24.3	13.7–34.8	30	75.0	61.6–88.4	130.1	15.9–244.3
	19–35 years	50	12.1	28	56.0	42.2–69.8	26.2	24.8–27.6	14	28.0	15.6–40.4	21.5	17.6–25.3	3	6.0	0–12.6	33.4	32.0–34.7	10	20.0	8.9–31.1	27.2	23.6–30.8	35	70.0	57.3–82.7	55.0	21.2–88.8
	36–55 years	231	56.1	138	59.7	53.4–66.1	26.6	25.8–27.4	49	21.2	15.9–26.5	19.2	17.3–21.0	31	13.4	9–17.8	28.0	25.9–30.2	50	21.6	16.3–27	25.6	24.3–27.0	174	75.3	69.8–80.9	51.8	36.7 −66.8
	> 55 years	66	16.0	37	56.1	44.1–68.0	24.2	22.9–25.5	14	21.2	11.3–31.1	21.0	17.9–24.0	9	13.6	5.4–21.9	28.1	24.8–31.4	12	18.2	8.9–27.5	26.0	23.2–28.9	53	80.3	70.7–89.9	44.8	28.8–60.8
Municipality	Palapag	293	71.1	161	54.9	49.2–60.6	26.6	25.9–27.3	73	24.9	20.3–30.2	19.4	18.0–20.9	27	9.2	6.4–1.3	28.6	26.5–30.6	62	21.2	16.9–26.2	26.4	25.2–27.6	212	72.4	67.0–77.3	49.7	36.3–63.1
	Laoang	119	28.9	81	68.1	59.2–75.8	25.2	24.3–26.0	17	14.3	9.1–21.7	23.0	18.7–27.2	23	19.3	13.2–27.3	29.4	26.7–32.0	17	14.3	9.1–21.7	24.1	20.8–28.4	95	79.8	71.7–86.1	93.4	50.0–136.7

*Abbreviations*: *CNI* gene copy number index, *Ct* Cycle threshold

^a^ddPCR analysis of faecal DNA. Information in this column is reproduced here from our previous publication (reference [40]) for comparison

### Prevalence of infections

**Fig. 2:**
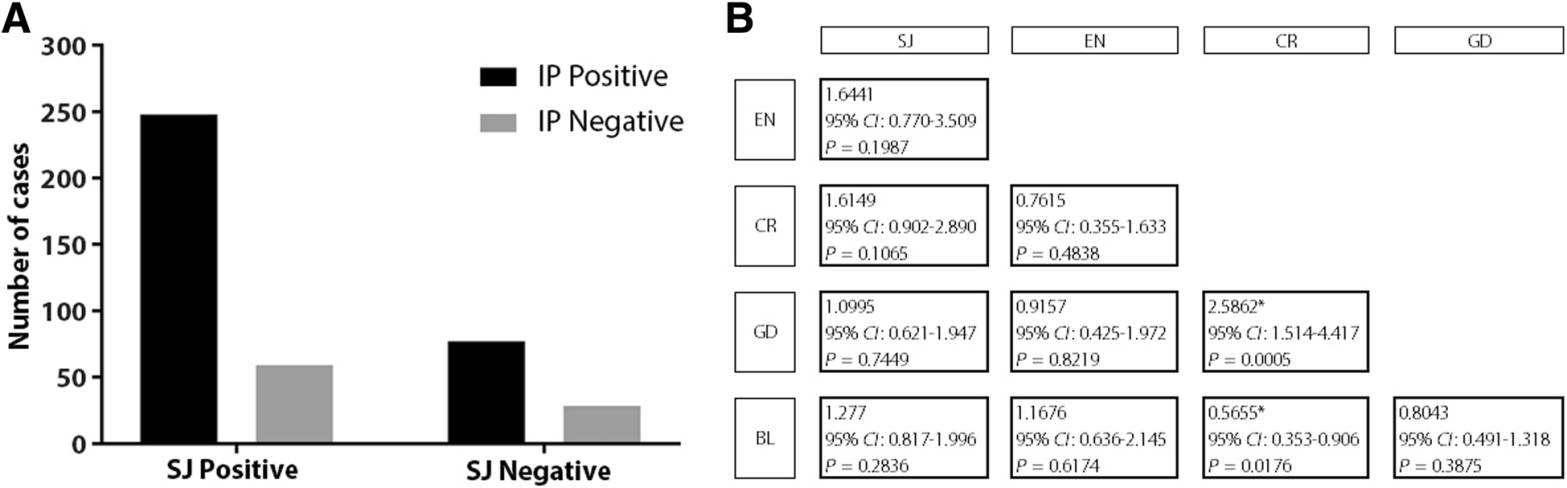
**a** Prevalence of IP (at least one co-infection) in subjects positive for *S. japonicum* infection compared with those *S. japonicum-*negative; **b** Odds ratio matrix showing associations between the four protozoans (BL, GD, CR and EN) and SJ infections. BL, *Blastocystis* spp.; CR, *Cryptosporidium parvum*; EN, *Entamoeba histolytica*; GD, *Giardia duodenalis*; SJ, *Schistosoma japonicum.* * Statistically significant associations

Prevalence was stratified by gender, age and municipality (Table 2). The prevalence of *S. japonicum* (74.5, 95% *CI:* 70.3–78.7) and *Blastocystis* spp. (58.7, 95% *CI:* 54.0*–*63.5) were significantly higher (*P* < 0.0001) compared to other infections (*Cryptosporidium* spp.; 21.8, 95% *CI:* 17.9*–*25.8, *G. duodenalis;* 19.2, 95% *CI:* 15.4*–*23.0, *E. histolytica;* 12.1, 95% *CI:* 9.0*–*15.3). The prevalence of *E. histolytica* was significantly lower than all other infections (*χ*^2^ = 536.6, *P* < 0.0001). Prevalence distribution of single and multiple parasite infections across different, age, gender and municipality categories are given in Table 3. There was no significant association of age or gender with different levels of co-infections. The majority of subjects who were infected with *S. japonicum* also had one or more IP co-infection (248/307, 80.8%) compared with individuals negative for *S. japonicum* (Fig. 2a), although the difference was not statistically significant (*χ*^2^ = 2.61, *P* ≥ 0.05).

**Table 3:** Prevalence of multiple co-infections with intestinal protozoa and *S. japonicum* by gender, age and municipality categories

Variables		Total		Single infection			Dual infection			Triple infection			Quadruple infection		
		*n*	%	Positive	Prevalence		Positive	Prevalence		Positive	Prevalence		Positive	Prevalence	
					%	95% *CI*		%	95% *CI*		%	95% *CI*		%	95% *CI*
Total study sample		412	100.0	115	27.9	23.6–32.2	175	42.5	37.7–47.2	76	18.4	14.7–22.2	15	3.6	1.8–5.4
Gender	Male	218	52.9	62	28.4	22.5–34.4	88	40.4	33.9–46.9	41	18.8	13.6–24.0	10	4.6	1.8–7.4
	Female	194	47.1	53	27.3	21.0–33.6	87	44.8	37.8–51.8	35	18.0	12.6–23.5	5	2.6	0.3–4.8
Age	< 10 years	25	6.1	11	44.0	24.5–63.5	6	24.0	7.3–40.7	5	20.0	4.3–35.7	0	0.0	0.0
	10–18 years	40	9.7	10	25.0	11.6–38.4	20	50.0	34.5–65.5	5	12.5	2.3–22.7	2	5.0	0.0–11.8
	19–35 years	50	12.1	15	30.0	17.3–42.7	19	38.0	24.5–51.5	8	16.0	5.8–26.2	2	4.0	0.0–9.4
	36–55 years	231	56.1	59	25.5	19.9–31.2	105	45.5	39.0–51.5	41	17.7	12.8–22.7	10	4.3	1.7–7.0
	> 55 years	66	16.0	20	30.3	19.2–41.4	25	37.9	26.2–49.6	17	25.8	15.2–36.3	1	1.5	0.0–4.5
Municipality	*Palapag*	293	71.1	89	30.4	25.4–35.9	119	40.6	35.1–46.3	51	17.4	13.5–22.2	13	4.4	2.6–7.5
	*Laoang*	119	28.9	26	21.8	15.4–30.1	56	47.1	38.3–56.0	25	21.0	14.7–29.2	5	4.2	1.8–9.5

**Fig. 3:**
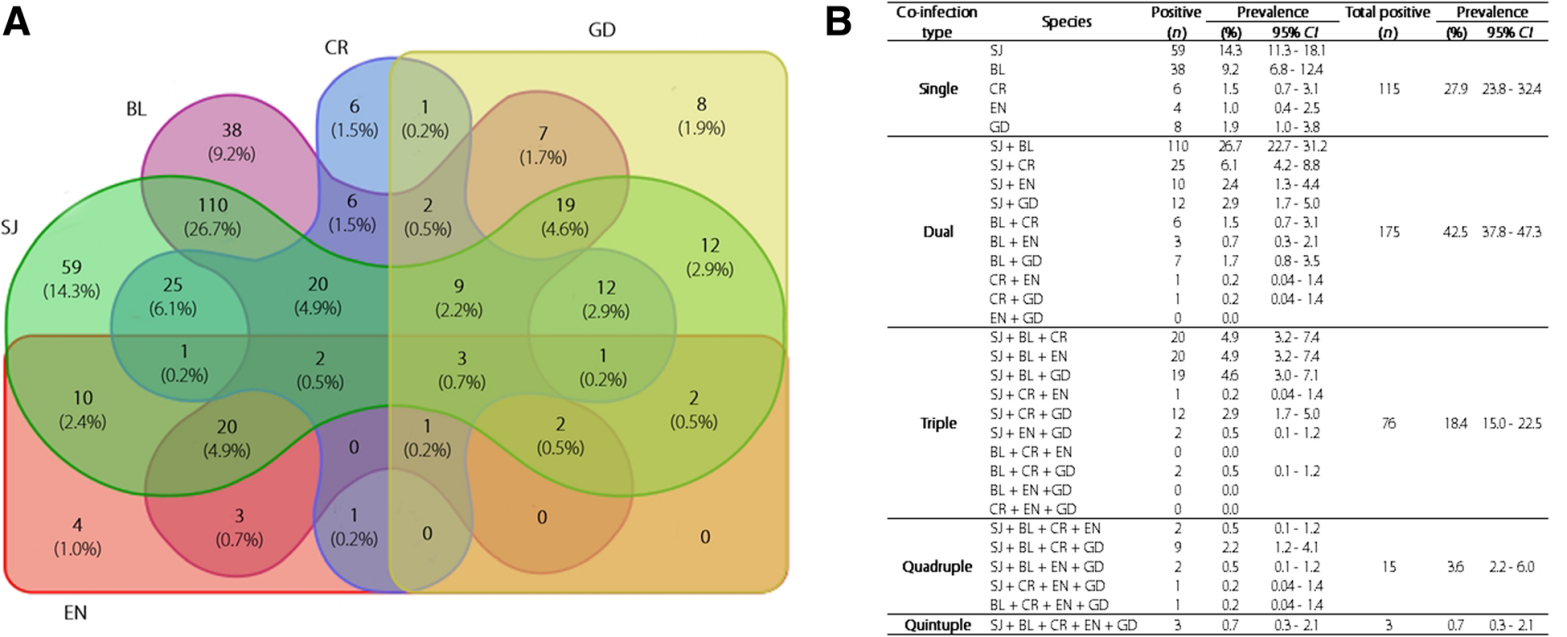
**a** Venn diagram showing the prevalence of single and multiple co-infections. **b** Prevalence of individual and multiple co-infection combinations. BL, *Blastocystis* spp.; CR, *Cryptosporidium parvum*; EN, *Entamoeba histolytica*; GD, *Giardia duodenalis*; SJ, *Schistosoma japonicum*

The majority of study participants had at least two infections (*n =* 269, 65.3%) (Table 3). The prevalence of dual infections was significantly higher than all other infections (*χ*^2^ = 185.2, *P* < 0.0001) (*n =* 175, 42.5, 95% *CI:* 37.7–47.2) and the prevalence of single infections (*n =* 115, 27.9, 95% *CI*: 23.6–32.2) was significantly higher than triple and quadruple infections (*χ*^2^ = 88.8, *P* < 0.0001). Of the dual infections, *S. japonicum* and *Blastocystis* were the most common combination (*n =* 110, 62.9%). Of the single infections 59 (14.3%) had schistosomiasis while 38 (9.2%) had *Blastocystis* infection (Fig. 3). Only three (0.73%) individuals (all female, aged between 34 and 54 years) harboured all five parasites. Of the total (*n =* 412), 28 (6.8%) were negative for all tested parasites. Of the school-aged children (age less than 18 years; *n =* 65), 58.5% (*n =* 38) had two or more infections and only six (9.2%) were negative for all tested parasites. Of women of child-bearing age (18–35 years; *n =* 23), 14 (60.1%) had at least two infections while only three (13.0%) had no infections.

### Coinfections with *S. japonicum*

*Blastocystis* was the most frequent co-infection occurring with *S. japonicum* (*n =* 185, 44.9%); *E. histolytica* was the least frequent (*n =* 41, 9.9%) (*χ*^2^ = 179.7, *P* < 0.0001). The co-infection prevalence of *Cryptosporidium* spp. and *G. duodenalis* with *S. japonicum* were 73 (17.7%) and 60 (14.6%), respectively (Fig. 3).

Of the single and multiple co-infection combinations, dual *S. japonicum* and *Blastocystis* spp. infection was the most prevalent (*n =* 110, 26.7%). Those with a frequency of at least 20 were *S. japonicum* alone (*n =* 59, 14.3%), *Blastocystis* spp. alone (*n* = 38, 9.2%), *S. japonicum* and *Cryptosporidium* spp. dual infection (*n =* 25, 6.1%), *S. japonicum*, *Blastocystis* spp. and *E. histolytica* triple infection (*n =* 20, 4.9%) and, *S. japonicum*, *Blastocystis* and *Cryptosporidium* spp. quadruple infection (*n =* 20, 4.9%) (Fig. 3).

### Associations of multiple infections

Calculated odds ratios showed cryptosporidiosis had a positive association with giardiasis (*OR*: 2.59, 95% *CI*: 1.51–4.42) and a negative association with *Blastocystis* spp. infection (*OR*: 0.57, 95% *CI*: 0.35–0.91). None of the other infections had significant associations with each other (Fig. 2b).

### Intensity of infection

Infection intensity of *S. japonicum* was significantly higher in Laoang municipality (copy number index [CNI] =93.4, 95% *CI*: 50.0–136.7, *P* < 0.05), and the intensity of *Blastocystis* spp. infection was significantly higher in Palapag municipality (Ct score = 26.6, 95% *CI*: 25.9–27.3, *P* < 0.05) (Table 2). The infection intensity of *Blastocystis* spp. varied significantly across different age categories (*P* < 0.005) (Table 2). Infection intensities of *S. japonicum* and *Blastocystis* spp. were significantly higher among males (*P* < 0.001). There were no significant differences in infection intensity with the other protozoan infections between males and females.

## Discussion

The current study reveals the extensive burden of multiple IP infections in the province of Northern Samar, a historically known endemic area for schistosomiasis [6, 32, 34, 40]. The majority of individuals in the study harboured at least two infections (*n =* 269, 65.3%) and only 28 (6.8%) had no infection at all. Moreover, the majority of subjects with *S. japonicum* infection were also co-infected with one or more IP (Fig. 2a). The most common individual parasites recorded were *S. japonicum* (74.5%) and *Blastocystis* spp. (58.7%). A very high prevalence of schistosomiasis japonica using advanced molecular diagnostics has previously been reported [5, 6, 40]. There is much continuing debate as to whether *Blastocystis* spp. is pathogenic or commensal in humans. This protozoan has been reported to give rise to gastrointestinal and dermatological manifestations but is common among infected individuals showing no clinical symptoms [23, 35, 42, 43]. Despite the fact that some patients experience symptoms such as diarrhoea, abdominal pain and bloating (which are mostly self-limiting) there are no confirmed virulent or pathogenic mechanisms explained in association with *Blastocystis* and it is generally termed a non-invasive organism [23, 42, 43]. However, it has also been reported that the elimination of heavy *Blastocystis* colonization results in symptom resolution. It is also considered that *Blastocystis* spp. may potentially play a role in the pathogenesis of chronic intestinal conditions such as irritable bowel syndrome [44, 45]. In contrast, the truly pathogenic protozoa *Entamoeba, Cryptosporidium* and *Giardia* present reported direct pathogenic effects such as red cell phagocytosis, disruption of gut mucosal epithelial cells and resultant diarrhoea and activation of immune responses [12, 20, 46]. The prevalence of *Cryptosporidium* spp. and *G. duodenalis* was significantly higher than that of *E. histolytica* in the region surveyed in our study. Individuals co-infected with these IPs have been reported globally with varying frequencies [47, 48].

In this study locality, cryptosporidiosis had a significant positive association with giardiasis but a negative association with blastocystosis (Fig. 2b), which contrasts with a study in rural Côte d’Ivoire, which reported a positive association between *Blastocystis* spp. and *G. duodenalis* [25]. *G. duodenalis* is believed to be mostly antagonistic (i.e. hindering infection by another parasite species) for concomitant infections [49, 50]. This association with *Giardia* is thought to arise either because of adverse physical interactions with other species or because of an abnormal overgrowth of bacteria in the jejunum [49]. A strong association between *G. duodenalis* and *S. mansoni* has been previously reported [28] and although no association was found between *S. japonicum* and *G. duodenalis* dual infection in the current study, we did record multiple IP co-infections with the schistosome. However, the prevalence of some parasites was low, which can affect the external validity of the strength of associations between different species.

There was no significant difference in the prevalence of any of the parasites in males and females in this study population. However, gender differences in the incidence and severity of parasitic diseases including schistosomiasis, IP and STH infections have been found previously [51]. This could mostly be attributed to gender-related behavioural differences, such as in occupational and household activities and/or to differences in the immune response amongst men and women, due to varying levels of sex hormones [51–53]. Over 60% of the females of child-bearing age had at least two infections while only 13% were negative for all tested parasites. This represents a potentially serious public health issue, particularly in relation to possible effects on pregnancy-associated complications such as intrauterine growth restriction and anaemia, leading to poor pregnancy outcomes [54]. Additionally, > 50% of the school-aged children harboured at least two parasite species and it is recognised that the chronic effects of these infections can have direct negative effects on growth, development and school performance [47, 55, 56].

**Fig. 4:**
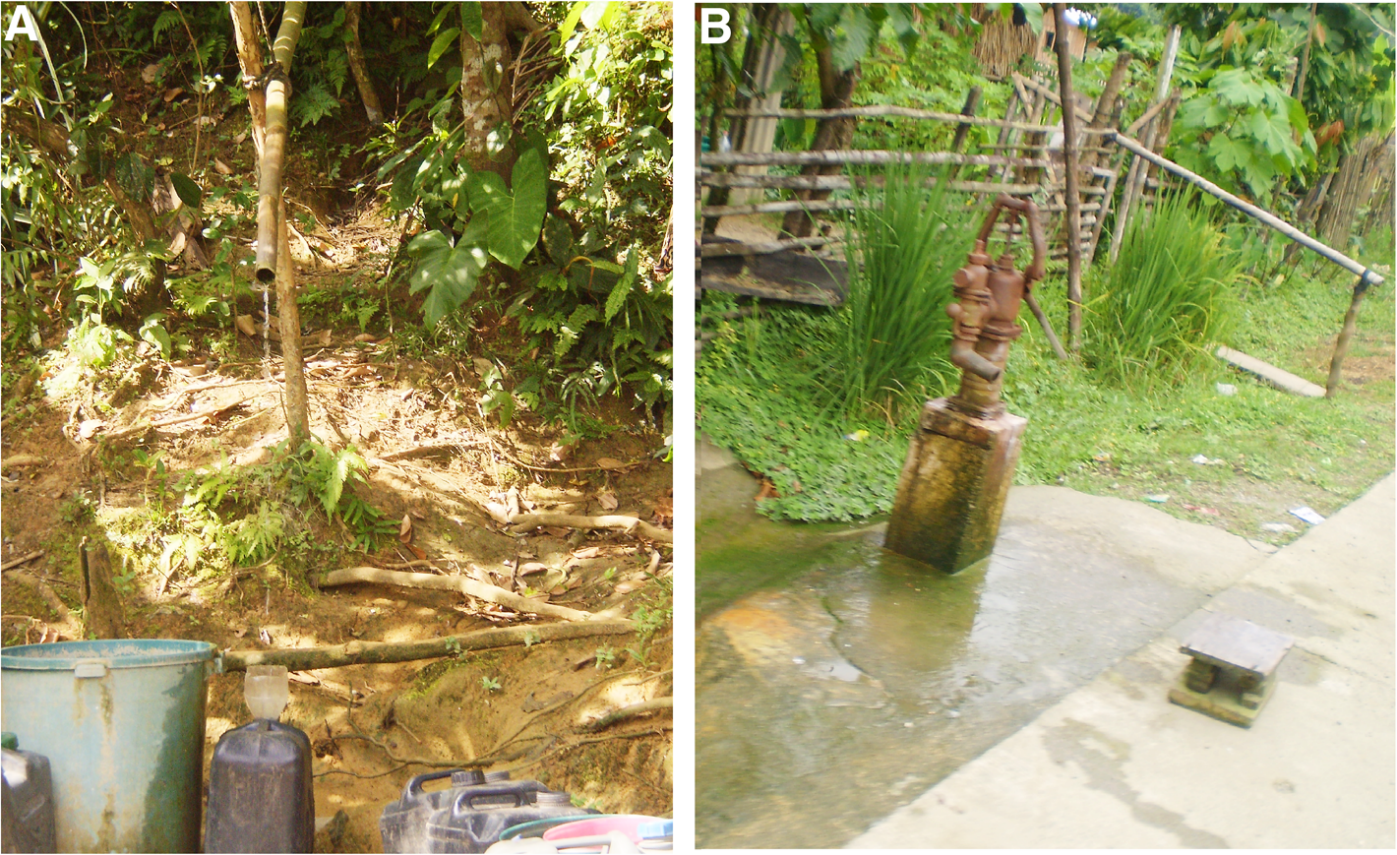
Some of the sources of water for human consumption in the study region: **a** Water directed from a spring through an open bamboo tube, **b** Well pump (depth approximately 5 ft)

The IPs infecting individuals from the study cohort share many aetiological and risk factors in common with schistosomes such as poor socio-economic status, a low level of education, limited access to safe water sources, poor sanitation and imperfect hygiene practices [33, 57, 58]. Hence the key control measures to target these parasites include improvement in access to clean water and sanitation, maintenance of good hygiene (WASH), health education, and mass drug administration (MDA) in endemic regions [33, 57–59]. The Laoang and Palapag municipalities are compromised in these respects in that villagers there mostly consume water from shallow wells, rivers and springs, which are exposed to water runoff and can easily be contaminated with sources such as waste water from leaking/poorly maintained septic systems (authors’ observations; Fig. 4), making it highly conducive to infection transmission and the likelihood of frequent co-infections [5, 33].

Schistosomiasis can result changes to local and systemic human host immunity, such as immune dysregulation with suppression of inflammatory Th1/17 responses. Moreover, schistosome-induced damage to the intestinal mucosa following egg entrapment and granuloma formation can pre-dispose the host to different IP infections and facilitate chronic protozoan infections [8, 28, 60–62]. Concomitant multiple parasite infections generally carry a higher morbidity compared with single infections. Multiple intestinal parasitic infections can cause additive or multiplicative effects on nutrition, immune status, growth and development, overall physical performance, and also increases the susceptibility to other types of gut pathogens such as cholera and rota virus [8, 28, 63–67]. Clinical manifestations of these chronic complications would mostly be accounted for by social factors such as poor socio-economic status and the protozoan infections may not be clinically suspected, unless the area is well known to be endemic for these infections. Without obvious acute symptoms such as diarrhoea and abdominal pain, and even with these symptoms, protozoan infections can easily be missed with commonly available low sensitivity copro-microscopic diagnostics [30, 68] leading to underlying chronic infections persisting with a higher long-term morbidity. It is therefore important to recognize clinical manifestations/patterns of infections of parasite communities, and to consider coinfections rather than just individual infections, all of which will help in early clinical suspicion and appropriate intervention.

It is imperative to use highly sensitive diagnostic tools required to detect asymptomatic infections through community screening so that efficient control and preventive measures can be implemented to target all parasites present in the endemic area. Both microscopic and molecular methods can detect multiple parasites in a single sample. However, copro-microscopic tests have the major drawbacks of being labour intensive, the need of multiple sample testing, the inability to differentiate between closely related species such as hookworms which are morphologically identical, and limited sensitivity particularly in low intensity infections [69, 70]. Hence PCR-based methods such as qPCR and ddPCR, particularly with their multiplexing capability, would be extremely helpful in accurate assessment of both prevalence as well as the intensity of co-parasite infections [30, 71–73].

While the PCR-based molecular assays are highly sensitive in disease diagnosis, they are relatively expensive and hence pose a major challenge for application as a routine diagnostic method and in screening campaigns. Adding to this, the need for advanced and specialized equipment leads to difficulties in establishing novel molecular technology directly in the field, mainly in resource-poor endemic regions. However, as the new technology matures and advances are made, these limitations, costs in particular, are likely to reduce. Despite the current limitations, PCR-based assays could be effectively applied as a monitoring tool for measuring the impact of control strategies by testing random community subsets as well as in secondary surveys for screening microscopy-negative individuals [5, 6, 40].

## Conclusions

Polyparasitism involving multiple IP infections and *S. japonicum* appears to be highly prevalent in rural Northern Samar province, likely contributing towards significant public health and socio-economic threats in this area and more generally throughout the Philippines. On a broader scale, the identification of individuals with polyparasitism using molecular tools, such as multiplex qPCR, is important as it can provide a measure of disease burden and also is a vital guide for the instigation of specific prevention and control interventions effective against multiple parasites.

## Additional file

Additional file 1: Multilingual abstracts in the five official working languages of the United Nations. (PDF 794 kb)

## Abbreviations

*CI*: Confidence interval
CNI: Copy number index
ddPCR: Droplet digital PCR
IP: Intestinal protozoa
MDA: Mass drug administration
*OR*: Odds ratio
PCR: Polymerase chain reaction
QIMR: Queensland Institute of Medical Research
QIMRB: QIMR Berghofer Medical Research Institute
qPCR: Quantitative PCR
STH: Soil transmitted helminths
WASH: Water, sanitation and hygiene
