# Supplementary material for: Co-parasitism of intestinal protozoa and Schistosoma japonicum in a rural community in the Philippines
Source: Infect Dis Poverty. 2018 Dec 10;7:121. doi: 10.1186/s40249-018-0504-6 (PMC6287361; doi:10.1186/s40249-018-0504-6)
Supplement: Supplementary file 1 — Multilingual abstracts in the five official working languages of the United Nations. (PDF 794 kb) [file 40249_2018_504_MOESM1_ESM.pdf]

Translation of the abstract into the six official working languages of the United Nations

طفيليات الحيوانات الأولية المعوية والبِلْهارْسِيَّةُ اليابانِيَّة في مجتمع ريفي في الفلبين

كوسالا ج. ويراكون، كاترين أ. غوردون، غيل م. ويليامز، بنغفي تساي ، جيفري ن. جوبيرت، ريميجيو م. أولفيدا، ألن ج. روس، ديفيد أ. أولفيدا، دونالد ب. مكمانوس

الملخص

خلفية: تعد الطفيليات ظاهرة متكررة في المجتمعات المحلية الفقيرة في المناطق الاستوائية وقد أسفرت عن عبء كبير من الأمراض . على الرغم من وجود تقارير مستفيضة عن داء الديداني المعوي، بما في ذلك مرض البلهارسيا اليابانية، إلا أن وجود أمراض تسببها الحيوانات الأولية المعوية ومداها لم تُدرس بعد بشكل عميق في الفلبين. نقدم تحليلاً مفصلاً لتعدد الطفيليات في مجتمع ريفي في سامار الشمالية، مع التركيز على تزامن الإصابة بالحيوانات الأولية المعوية مع البِلْهارْسِيَّةُ اليابانِيَّة.

أساليب: أجريت دراسة مقطعية وصفية عام 2015 في 18 قرية تعاني من البِلْهارْسِيَّةُ اليابانِيَّة في شمال سامار في الفلبين لتقييم عبء مرض البلهارسيا البشرية وإصابات الحيوانات الأولية المعوية. وقد تضمن التحليل الجزيئي النهائي عينات براز أُخذت من 412 مشارك من 18 قرية. كما أُعدت مقالة عن تفاعل البلمرة المتسلسل الكمي المتعدد واستُخدمت للكشف عن *المُتبرْعمةُ الكيسيةو المُتَحَوّلَةُ الحالَّةُ للنُّسُج* و كريبتوسبوريديوم *(خفية الأبواغ) و الجياردية المعوية* في عينات البراز. تم الجمع بين هذه النتائج و نتائج سابقة لتشخيصات رقمية تقطيرية لتفاعل البلمرة المتسلسل لدى أفراد من نفس الـ 18 قرية المصابة بالبلهارسية اليابانية كما أُستخدمت نفس عينات البراز للتحليل .

النتائج: متوسط عمر المشاركين في الدراسة كان 40.3 سنة (95٪ مجال الثقة: 38.8-) بنسبة 53٪ (ن = 218) من الذكور. انتشار البلهارسية اليابانية بنسبة (74.5 و المتبرعمة الكيسية بنسبة(58.7٪) كانت أعلى بشكل ملحوظ مقارنة بالعدوى الأخرى، كما أن انتشار المُتَحَوّلَةُ الحالَّةُ للنُّسُجكان الأدنى بمعدل (12.1 ٪). مجموعة كبيرة من الأفراد كانوا مصابين بأكثر من طفيلي واحد مع وجود مرضين هم الأكثر شيوعاً (n = 175، 42.5 %). كان معدل انتشار إصابة الأفراد بطفيليين اثنين أعلى بقليل من الـ 27.9% (n = 115) من الأفراد المصابين بنوع واحد من الطفيليات. كان الجمع بين مرضي البلهارسيات اليابانيةو المتبرعمة الكيسية هي التركيبة الأكثر شيوعاً بين الأفراد (n = 110، 62.9 %). بعد دراسة عامل العمر بين السكان، تبين إصابة 58.5 في المائة (n = 38) من الأطفال ممن هم في سن المدرسة و 60.1% (n = 14) من النساء في سن الحمل بنوعين على الأقل من الطفيليات.

الاستنتاجات: كشفت الدراسة انتشار تعدد الطفيليات مع عدوى الحيوانات الأولية المعوية وداء البلهارسيا اليابانية بشكل كبير بين الأفراد في شمال سامار مما يساهم على الأرجح في الأعباء الاقتصادية والاجتماعية والصحية التي يعانيها هؤلاء السكان. وبصورة أعم، تعد هذه النتائج ذات أهمية عند النظر في تنفيذ استراتيجيات مكافحة متكاملة للطفيليات المعوية.

Translated from English version into Arabic by Ghada Alluhaidan, proofread by Malika, through

TRANSLATORS
WITHOUT BORDERS

菲律宾农村社区肠道原虫和日本血吸虫合并感染情况

Kosala G. Weerakoon, Catherine A. Gordon, Gail M. Williams, Pengfei Cai, Geoffrey N. Gobert, Remigio M. Olveda, Allen G. Ross, David U. Olveda, Donald P. McManus

摘要

引言：一人同时感染两种或几种寄生虫在热带贫困社区很常见，造成严重的疾病负担。在菲律宾，虽然有大量关于肠道蠕虫病包含日本血吸虫病在内的报道，但尚未对肠道原虫引起的疾病情况进行深入研究。本研究详细分析了菲律宾萨马尔北部农村社区的多虫感染情况，着重于肠道原虫与日本血吸虫的合并感染。

方法：2015 年在菲律宾萨马尔北部 18 个日本血吸虫病流行村开展描述性横断面研究，以评估人类血吸虫病和肠道原虫感染的疾病负担。从 18 个村庄收集的 412 名参与者的粪便样本用于分子生物学分析。采用多重定量 PCR 法检测粪便样本中的芽囊原虫（*Blastocystis* spp.）、溶组织内阿米巴（*Entamoeba histolytica*）、隐孢子虫（*Cryptosporidium* spp.）和十二指肠贾第鞭毛虫（*Giardia duodenalis*），并将其检测结果与先前使用相同粪样以液滴式数字 PCR 法检测日本血吸虫感染情况相结合进行诊断。

结果：研究参与者的平均年龄为 40.3 岁（95% *CI*: 38.8-41.8），其中 53%（$n = 218$）)为男性。日本血吸虫（74.5%）和芽囊原虫（58.7%）的感染率明显高于其他感染，溶组织内阿米巴（12.1%）的感染率最低。大多数人感染一种以上寄生虫，最常见的是感染两种（$n = 175$，42.5%）。同时感染两种寄生虫的个体显著高于单一感染的个体（$n = 115$，27.9%）。同时感染两种寄生虫的个体中，日本血吸虫和芽囊原虫是最常见的组合($n = 11$，62.9%)。学龄儿童 ($n =$ 14，58.5%)和育龄妇女($n = 38$，60.1%)至少携带两种寄生虫。

结论：本研究表明，在菲律宾萨马尔北部地区肠道原虫和日本血吸虫病的多虫感染非常普遍，可能导致严重的公共卫生和社会经济负担。因此，在实施肠道寄生虫综合控制策略时，需考虑多虫感染的情况。

Translated from English version into Chinese by Hao-Nan Wu, edited by Pin Yang

CHINESE CENTER FOR DISEASE CONTROL AND PREVENTION
NATIONAL INSTITUTE OF PARASITIC DISEASES

Co-infection par des protozoaires intestinaux et *Schistosoma japonicum* dans une communauté rurale aux Philippines

Kosala G. Weerakoon, Catherine A. Gordon, Gail M. Williams, Pengfei Cai, Geoffrey N. Gobert, Remigio M. Olveda, Allen G. Ross, David U. Olveda, Donald P. McManus

Résumé

Contexte: Les co-infections parasitaires sont fréquentes au sein des populations pauvres des régions tropicales et constituent un fardeau considérable de morbidité. Alors que les helminthiases intestinales, notamment la schistosomiase sino-japonaise, ont déjà fait l'objet d'abondants rapports, la présence et l'ampleur des maladies causées par des protozoaires intestinaux n'ont pas encore été étudiées de manière approfondie aux Philippines. Nous présentons ici une analyse détaillée des polyparasitoses dans une communauté rurale de la province de Samar du Nord, et en particulier des co-infections par des protozoaires intestinaux et *Schistosoma japonicum*.

Méthodes: Une étude transversale descriptive a été réalisée en 2015 dans 18 barangays (villages) où *S. japonicum* était endémique dans la province de Samar du Nord, aux Philippines. Le but était d'évaluer le fardeau de la schistosomiase humaine et des protozooses intestinales. Les échantillons de selles de 412 participants ont été recueillis dans les 18 barangays et inclus dans l'analyse moléculaire finale. Un

essai par RCP quantitative multiplex a été mis au point et utilisé pour détecter *Blastocystes* spp., *Entamoeba histolytica*, *Cryptosporidium* spp. et *Giardia duodenalis* dans ces échantillons. Les résultats ont été combinés aux résultats antérieurs de diagnostic numérique de goutelettes par RCP sur ponction digitale d'habitants de ces mêmes barangays infectés par *S. japonicum*, sur la base des mêmes échantillons de selles.

Résultats: L'âge moyen des participants à l'étude était de 40,3 ans (*IC* à 95% de 38,8 à 41,8) et 53% (*n* = 218) étaient de sexe masculin. La prévalence de *S. japonicum* (74,5%) et *Blastocystis* spp.(58,7 %) était significativement plus élevée que celle d'autres parasites, la plus faible prévalence étant celle d'*E. histolytica* (12,1%). La majorité des sujets étaient porteurs de plus d'un parasite, l'association de deux infections étant la plus fréquente (*n* = 175, 42,5 %). La prévalence des porteurs de deux parasites était significativement plus élevée que celle de tous les autres cas, 27,9 % (*n* = 115) des sujets étant porteurs d'une seule espèce. Parmi les sujets infectés par deux parasites, la combinaison la plus fréquente était celle de *S. japonicum* et *Blastocystis* spp. (*n* = 110, 62,9 %). L'examen de l'âge dans cette population montre que 58,5 % (*n* = 38) des enfants d'âge scolaire et 60,1% (*n* = 14) des femmes en âge de procréation étaient porteuses d'au moins deux espèces de parasites.

Conclusions: Cette étude a révélé une prévalence élevée des protozooses intestinales associées à Schistosoma japonicum parmi la population de la province de Samar du Nord. Ces co-infections parasitaires contribuent probablement au fardeau sanitaire et socioéconomique important qui pèse sur cette population. Plus généralement, ces observations sont pertinentes dans l'optique du déploiement de stratégies intégrées de lutte contre les parasites intestinaux.

Translated from English version into French by Suzanne Assenat, proofread by Djeukeu Kana Christelle, through

TRANSLATORS
WITHOUT BORDERS

Совместное заражение простейшими кишечными паразитами и *Schistosoma japonicum* у сельских жителей на Филиппинах

Косала Дж. Виракун, Кэтрин А. Гордон, Гейл М. Уильямс, Пенгфей Цай, Джеффри Н. Гоберт, Ремихио М. Ольведа, Аллен Дж. Росс, Дэвид Ю. Ольведа, Дональд П. МакМанус

Аннотация

Справочная информация: Совместное заражение паразитами часто встречается в бедных поселениях в тропиках, что приводит к высоким показателям заболеваемости. Несмотря на значительный объём данных о заболеваемости кишечным гельминтозом, включая японский шистосомоз, необходимо всестороннее исследование возникновения и масштабов заболеваний, вызванных простейшими кишечными паразитами (ПКП) на Филиппинах. Мы представляем подробный анализ полипаразитизма в поселениях провинции Северный Самар, уделяя первоочередное внимание совместным заражениям ПКП и паразитом *Schistosoma japonicum*.

Методы: Для оценки заболеваемости шистосомозом и инфицирования ПКП в 2015 году проводилось описательное поперечное исследование в 18 эндемичных для *S. japonicum* барангаях (поселениях) провинции Северный Самар (Филиппины). Пробы кала, взятые у 412 участников из 18 барангаев, были исследованы в рамках заключительного молекулярного анализа. Мультиплексный количественный анализ ПЦР был разработан и применён для обнаружения в пробах кала видов *Blastocystis, Entamoeba histolytica*, *Cryptosporidium* и *Giardia duodenalis.* Эти данные были объединены с ранее полученными результатами цифровой капельной ПЦР проб, взятых у лиц из тех же 18 барангаев, зараженных *S. japonicum,* диагностированных с использованием тех же проб кала.
Результаты: Средний возраст участников исследования был 40,3 года (95% *CI:* 38,8–41,8), причём 53% (*n* = 218) среди них составляли мужчины. Распространённость видов *S. japonicum* (74,5%) и *Blastocystis* (58,7%) была значительно выше по сравнению с другими инфекциями, причём *E. histolytica* отличался самой низкой распространённостью (12,1%). Большинство лиц были инфицированы более чем одним паразитом, при этом наиболее распространены были две инфекции (*n* = 175; 42,5%). Показатель заражённости лиц двумя видами паразитов был значительно выше, чем все прочие показатели. Было также выявлено, что 27,9% (*n* = 115) участников исследования являлись переносчиками одного вида паразита. У лиц, инфицированных двумя паразитами, виды *S. japonicum* и *Blastocystis* были наиболее распространенной комбинацией (*n* = 110; 62,9%). Анализ возраста населения показал, что 58,5% (*n* = 38) детей школьного возраста и 60,1% (*n* = 14) женщин детородного возраста являются переносчиками по крайней мере двух видов паразитов.
Выводы: Исследование показало, что полипаразитизм с одновременным заражением инфекциями ПКП и японским шистосомозом очень распространен среди населения провинции Северный Самар, что вероятно способствует возникновению существенных проблем в сферах общественного здравоохранения и социально-экономического развития среди этих категорий населения. В более общем смысле, результаты исследования являются значимыми при рассмотрении вопроса о реализации интегрированных стратегий борьбы с заражением кишечными паразитами.

Translated from English version into Russian by Galina Dmitrieva, proofread by Natalia Potashnik, through

TRANSLATORS WITHOUT BORDERS

Coparasitismo de protozoos intestinales y *Schistosoma japonicum* en una comunidad rural en Filipinas

Kosala G. Weerakoon, Catherine A. Gordon, Gail M. Williams, Pengfei Cai, Geoffrey N. Gobert, Remigio M. Olveda, Allen G. Ross, David U. Olveda, Donald P. McManus

Resumen

Introducción: El coparasitismo es un hecho frecuente en comunidades tropicales empobrecidas que provoca una carga considerable de enfermedad. A pesar de que existen informes exhaustivos sobre helmintiasis intestinal, como la esquistosomiasis japónica, aún no se ha investigado detalladamente la ocurrencia y el alcance de las enfermedades causadas por protozoos intestinales (PI) en Filipinas. Presentamos un análisis detallado de poliparasitismo en una comunidad rural en Sámar del Norte, enfocado en las coinfecciones de PI con *Schistosoma japonicum*.

Métodos: Se llevó a cabo un estudio trasversal descriptivo en 2015 a lo largo de 18 barangayes (barrios) endémicos de *S. japonicum* en Sámar del Norte, Filipinas para evaluar la carga de esquistosomiasis humana e infecciones PI. Se incluyeron en el análisis molecular final muestras fecales recolectadas de 412 participantes de los 18 barangayes. Se desarrolló y usó un ensayo de PCR múltiple cuantivativa para la detección de *Blastocystis* spp., *Entamoeba histolytica*, *Cryptosporidium* spp. y *Giardia duodenalis* en las muestras fecales. Los hallazgos se combinaron con resultados previos de diagnóstico PCR digital por gotículas de individuos procedentes de los mismos 18 barangayes infectados con *S. japonicum* que fueron determinados con las mismas muestras fecales disponibles para el análisis.

Resultados: La edad promedio de los participantes del estudio fue 40.3 años (95 % *IC:* 38.8 - 41.8), de los cuales un 53 % (*n* = 218) eran hombres. La prevalencia de *S. japonicum* (74.5 %) y *Blastocystis* spp. (58.7 %) fue considerablemente mayor en comparación con otras infecciones, como *E. histolytica* que tuvo la menor prevalencia (12.1 %). La mayoría de los individuos estaban infectados por más de un parásito y lo más frecuente era tener dos infecciones (*n* = 175, 42.5 %). La prevalencia de individuos con dos parásitos fue considerablemente mayor que los demás con un 27.9 % (*n* = 115) de sujetos albergando una sola especie de parásito. Entre los individuos con dos infecciones, *S. japonicum* y *Blastocystis* spp. fue la combinación más frecuente (*n* = 110, 62.9 %). Al examinar la edad de la población, un 58.5 % (*n* = 38) de los niños en edad escolar y un 60.1 % (*n* = 14) de las mujeres en edad fértil albergaban al menos dos especies de parásitos.

Conclusiones: El estudio demostró que el poliparasitismo con infecciones PI y la esquistosomiasis japónica es sumamente prevalente en individuos en Sámar del Norte, lo que probablemente contribuye al problema de salud pública y socioeconómico que sufre esta población. En un sentido más amplio, estos resultados son pertinentes cuando se considera la implementación de estrategias de control integrado de parásitos intestinales.

Translated from English version into Spanish by Julieta Gaitan, proofread by Ana Leticia Santos, through

TRANSLATORS
WITHOUT BORDERS
